# Bis(2,2′-bipyridine-κ^2^ *N*,*N*′)(maleato-κ^2^ *O* ^1^,*O* ^1′^)nickel(II) 7.34-hydrate

**DOI:** 10.1107/S1600536808036672

**Published:** 2008-11-13

**Authors:** Anna Pavlová, Juraj Černák, Klaus Harms

**Affiliations:** a Department of Inorganic Chemistry, Institute of Chemistry, P. J. Šafárik University, Moyzesova 11, 041 54 Košice, Slovakia; b Fachbereich Chemie der Philipps-Universität Marberg, Hans-Meerwein Strasse, D-35032 Marburg, Germany

## Abstract

The title compound, [Ni(C_4_H_2_O_4_)(C_10_H_8_N_2_)_2_]·7.34H_2_O, was obtained by crystallization from an aqueous ethano­lic reaction mixture containing nickel(II) acetate, maleic acid, bipyridine, sodium hydroxide and ammonia. The asymmetric unit contains one independent complex mol­ecule and 7.34 water mol­ecules occupying eight crystallographically independent positions. Two of these water molecules are disordered. The nickel(II) atom is coordinated in a distorted octa­hedral geometry by two O atoms from one carboxyl­ate group of the maleato ligand and by four N atoms from two 2,2′-bipyridine (bipy) ligands. The water mol­ecules, along with the O atoms of the uncoordinated carboxyl­ate group, form an extended hydro­philic three-dimensional hydrogen-bonded system with large cavities in which the hydro­phobic bipy ligands are located. One H atom of the maleate ligand is involved in a weak hydrogen bond of the C—H⋯O type. Stacking inter­actions between the pyridyl rings of the bipy ligands [centroid–centroid distance = 3.549 (15) Å] lead to the formation of pairs of complex mol­ecules.

## Related literature

For magnetic studies of nickel(II) complexes, see: Boča (2004 ▶); Kamieniarz *et al.* (2007 ▶); Paharová *et al.* (2003 ▶); Černák *et al.* (2003 ▶). Several complexes containing the [Ni(bipy)_2_]^2+^ structural motif completed with various anionic ligands including acetato (Holz *et al.*, 1996 ▶), oxalato (Roman *et al.*, 1995 ▶) and terephtalato (Deng *et al.*, 1992 ▶) have been structurally characterized. The maleato ligand can act as a monodentate (Sequeira *et al.*, 1992 ▶), bidentate (Zheng & Kong, 2003 ▶), tridentate (Xue *et al.*, 2005 ▶) or tetra­dentate (Chen *et al.*, 2003 ▶) ligand. For the crystal structure of the similar [Ni(bipy)(mal)(H_2_O)_3_]·H_2_O complex, see: Li *et al.* (2006 ▶). For [Ni(dpa)_2_(suc)_0.5_]Cl (dpa = 4,4′-dipyridylamine, suc = succinato dianion), which has similar geometric parameters and a similar type of coordination to the title compound, see: Montney *et al.* (2007 ▶). The maleato ligand in {[Zn(H_2_O)_4_(*L*_1_)Zn(mal)_2_]·H_2_O}_*n*_, [*L*_1_ = N-(3-pyrid­yl)-isonicotinamide] has a similar coordination, see: Kumar *et al.* (2006 ▶).
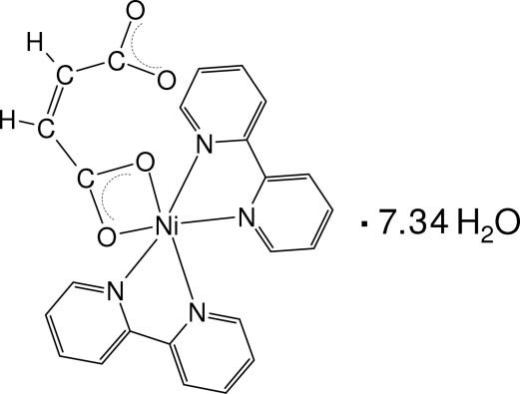


## Experimental

### 

#### Crystal data

[Ni(C_4_H_2_O_4_)(C_10_H_8_N_2_)_2_]·7.34H_2_O
*M* *_r_* = 617.35
Monoclinic, 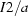

*a* = 20.7108 (6) Å
*b* = 17.4754 (5) Å
*c* = 15.6460 (6) Å
β = 97.767 (3)°
*V* = 5610.8 (3) Å^3^
*Z* = 8
Mo *K*α radiation
μ = 0.76 mm^−1^
*T* = 100 (2) K
0.36 × 0.18 × 0.16 mm

#### Data collection

Stoe IPDS diffractometer
Absorption correction: multi-scan (Blessing, 1995 ▶) *T*_min_ = 0.772, *T*_max_ = 0.888
14173 measured reflections
4944 independent reflections
4176 reflections with *I* > 2σ(*I*)
*R*_int_ = 0.024

#### Refinement

*R*[*F* ^2^ > 2σ(*F* ^2^)] = 0.023
*wR*(*F* ^2^) = 0.058
*S* = 0.96
4944 reflections
422 parameters
8 restraints
H atoms treated by a mixture of independent and constrained refinement
Δρ_max_ = 0.26 e Å^−3^
Δρ_min_ = −0.24 e Å^−3^

### 

Data collection: *X-AREA* (Stoe & Cie, 2007 ▶); cell refinement: *X-AREA*; data reduction: *X-AREA*; program(s) used to solve structure: *SHELXS97* (Sheldrick, 2008 ▶); program(s) used to refine structure: *SHELXL97* (Sheldrick, 2008 ▶); molecular graphics: *DIAMOND* (Brandenburg, 2006 ▶); software used to prepare material for publication: *SHELXL97*.

## Supplementary Material

Crystal structure: contains datablocks I, global. DOI: 10.1107/S1600536808036672/rz2262sup1.cif

Structure factors: contains datablocks I. DOI: 10.1107/S1600536808036672/rz2262Isup2.hkl

Additional supplementary materials:  crystallographic information; 3D view; checkCIF report

## Figures and Tables

**Table 1:** Hydrogen-bond geometry (Å, °)

*D*—H⋯*A*	*D*—H	H⋯*A*	*D*⋯*A*	*D*—H⋯*A*
O5—H51⋯O6	0.79 (2)	1.99 (3)	2.7864 (16)	178 (3)
O5—H52⋯O3^i^	0.86 (3)	1.88 (3)	2.7466 (17)	177 (2)
O6—H61⋯O12^ii^	0.84 (2)	1.90 (2)	2.7384 (16)	174 (3)
O7—H71⋯O8^iii^	0.8500 (11)	1.997 (3)	2.844 (2)	174 (2)
O7—H72⋯O9^iv^	0.8500 (10)	1.996 (4)	2.8388 (19)	171 (2)
O8—H81*A*⋯O1	0.8500 (10)	2.539 (14)	3.3576 (18)	162 (4)
O8—H82⋯O5^v^	0.850 (9)	1.991 (9)	2.8406 (17)	178 (2)
O8—H81*B*⋯O8^vi^	0.8500 (11)	2.084 (11)	2.918 (3)	167 (4)
O9—H91⋯O4	0.89 (2)	1.87 (3)	2.7516 (16)	173 (2)
O9—H92⋯O1^vii^	0.82 (2)	1.96 (3)	2.7701 (16)	173 (2)
O10—H101⋯O3	0.82 (3)	1.88 (3)	2.6905 (17)	168 (3)
O10—H102⋯O11^viii^	0.86 (3)	1.89 (3)	2.7409 (19)	177 (3)
O11—H111⋯O5^ix^	0.83 (3)	2.03 (3)	2.8333 (19)	161 (2)
O11—H112⋯O9^viii^	0.85 (3)	1.95 (3)	2.7752 (18)	162 (2)
O12—H121⋯O10^viii^	0.79 (3)	2.00 (3)	2.7844 (18)	172 (2)
O12—H122⋯O10	0.82 (3)	1.97 (3)	2.7839 (18)	170 (2)
C23—H18⋯O7^vii^	0.95	2.38	3.288 (2)	159

Symmetry codes: (i) 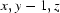; (ii) 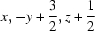; (iii) 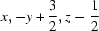; (iv) 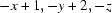; (v) 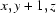; (vi) 
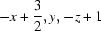
; (vii) 
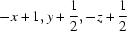
; (viii) 
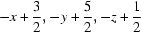
; (ix) 
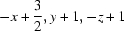
.

**Fig. 1.**
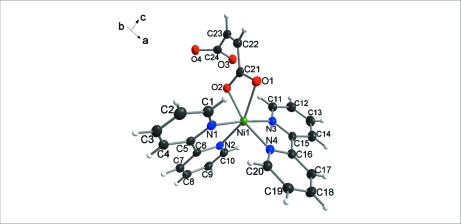
The molecular structure of the title complex with the atom-numbering scheme. Displacement ellipsoids are drawn at the 50 % probability level. Water molecules are omitted for clarity.

**Fig. 2.**
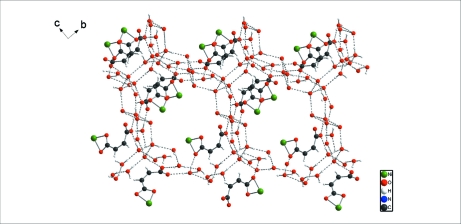
View of the hydrogen bonding system (dashed lines) showing formation of large cavities which accommodate the hydrophobic parts of the complex molecules. Only one disordered position for the hydrogen atoms bound to O8 atom is shown. For the sake of clarity, only the N donor atoms of the bipy ligands are shown.

**Fig. 3.**
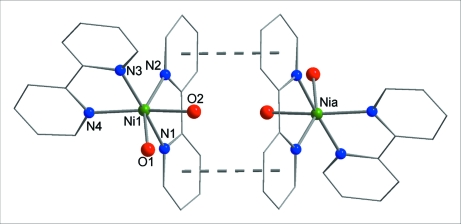
View of the π-π interactions (dashed lines) occurring between the aromatic rings of adjacent bipy ligands. Only the oxygen atoms of the maleato ligands are shown. Hydrogen atoms are omitted for clarity. Symmetry code: (a) 1.5-x, y, -z.

## supplementary crystallographic information

### Comment

Previously we have studied the crystal structures and the structure-magnetic properties correlations of several low-dimensional cyanocomplexes in which the paramagnetic nickel(II) central atoms were bridged by suitable cyanocomplex anions (Černák *et al.*, 2003; Paharová *et al.*, 2003). As a continuation of our studies we started to investigate low-dimensional systems of nickel(II) with the same type of N-donor blocking ligands, but using dicarboxylate anions as bridging species in place of cyanocomplex anions. As a result of our synthetic effort, we have isolated the title compound, [Ni(bipy)_2_(mal)].7,34H_2_O, and here we report its crystal structure. The crystal structure of the similar [Ni(bipy)(mal)(H_2_O)_3_].H_2_O complex was recently reported (Li *et al.*, 2006).

The crystal structure of the title compound is molecular (Fig. 1). The complex molecule is formed by an octahedrally-distorted coordinated nickel(II) atom to which two chelate bonded bipy ligands and one chelate bonded maleato ligand are coordinated, thus the chromophore is of the cis-NiN_4_O_2_ type. As can be seen from the values of the bond angles, the octahedron around the nickel atom is rather deformed; the O1-Ni-O2 angle within the four-membered chelate ring formed by the carboxylate group of the maleato ligand is rather acute (61.86 (4)°) while the widest angle is O2-Ni-N4 (164.87 (5)°). The Ni-N bond lengths vary between 2.0461 (13) and 2.0608 (13) Å, and the Ni-O bond lengths exhibit values of 2.0935 (10) and 2.1678 (11) Å. Such type of coordination was already observed in [Ni(dpa)_2_(suc)_0.5_]Cl (dpa = 4,4'-dipyridylamine, suc = succinato dianion) with similar geometric parameters (Montney *et al.*, 2007). Both bipy ligands are planar. The maleato ligand acts as a bidentate chelate bonded ligand with an uncoordinated carboxylate group. To our knowledge, such coordination of the maleato ligand was previously observed only in {[Zn(H_2_O)_4_(L_1_)Zn(mal)_2_].H_2_O}_n_, (L_1_ = N-(3-pyridyl)-isonicotinamide; Kumar *et al.*, 2006). The observed geometric parameters associated with the ligands in the title compound do not show unusual features.

In the unit cell there are eight crystallographically independent positions occupied by the oxygen atoms of the crystal water molecules. The O6 oxygen atom lies on a twofold rotation axis and the O7 oxygen atom exhibits a partial occupancy of 0.84, thus there are 7.34 water molecules per formula unit. These water molecules along with the oxygen atoms of the uncoordinated carboxylate group form an extended hydrophilic three-dimensional hydrogen bonded systems with O···O distances ranging from 2.6905 (17) to 3.3576 (18) Å (Table 1), leading to the formation of large cavities (Fig. 2) in which the bipy ligands are situated. To the hydrogen bonding network contributes also the weak C—H···O hydrogen bond (Table 1). Between pairs of bipy ligands π-π stacking interactions operate (Fig. 3). The centroid···centroid distance between the aromatic rings (3.549 (15) Å) is similar to that found in [Ni(bipy)_2_(ox)].4H_2_O (ox = oxalato dianion). These interactions lead to formation of pairs of complex molecules (Fig. 3).

### Experimental

Chemicals were of reagent grade quality obtained from commercial sources and were used as received without further purification. All solutions were prepared by using deionized water and ethanol (96 % v.v.). To 5 cm^3^ of an aqueous solution of Ni(CH_3_COO)_2_.4H_2_O (0.4974 g, 2 mmol) was added under stirring at room temperature a previously prepared solution containing 2,2'-bipyridine (0.624 g, 4 mmol) and maleic acid (0.232 g, 2 mmol) in ethanol. To the formed clear violet solution were further added NaOH (0.160 g, 4 mmol) dissolved in water (4 cm^3^) and a  concentrated (25 % v.v.) aqueous solution of ammonia (5 cm^3^). The formed red-violet solution was stirred for 3 minutes at 90 °C and was left to evaporate slowly at room temperature. After one week, few violet plates of the title compound appeared; one of them was picked off for X-ray structure analysis. After disturbing the mother liquor, immediate jellification started.

### Refinement

The O7 atom exhibited larger thermal ellipsoid at full occupancy than the other water oxygen atoms, so its occupancy was refined to a value of 0.841 (5); in the final cycles of refinement this value was fixed at 0.84. The hydrogen atoms of the water molecules were located in difference map. Their positions were refined with common isotropic thermal parameter for the pair of hydrogen atoms of the same molecule. This was not the case for the hydrogen atoms bound to the O7 oxygen atom with partial site occupancy as well as for the hydrogen atoms bound to O8 atom as two disordered positions with occupancy 0.5 were found; the disorder is the consequence of close position of the O8 atom and its symmetry equivalent at 1.5-x, y, 1-z. The positions of the hydrogen atoms bound to O7 and O8 atoms were constrained by geometric parameters, with values of 0.850 (1) and 1.344 (1) Å for the O—H and H···H distances, respectively.  All other hydrogen atoms were positioned geometrically and constrained to ride on their parent atoms, with C—H = 0.95 Å and with U_iso_(H) = 1.2U_eq_(C).

### Crystal data

**Table:**

[Ni(C_4_H_2_O_4_)(C_10_H_8_N_2_)_2_]·7.34H_2_O	*F*_000_ = 2587.2
*M**_r_* = 617.35	*D*_x_ = 1.462 Mg m^−^^3^
Monoclinic, *I*2/*a*	Mo *K*α radiation λ = 0.71073 Å
Hall symbol: -I 2ya	Cell parameters from 16806 reflections
*a* = 20.7108 (6) Å	θ = 3.1–54.4º
*b* = 17.4754 (5) Å	µ = 0.76 mm^−^^1^
*c* = 15.6460 (6) Å	*T* = 100 (2) K
β = 97.767 (3)º	Plate, violet
*V* = 5610.8 (3) Å^3^	0.36 × 0.18 × 0.16 mm
*Z* = 8	

### Data collection

**Table:**

Stoe IPDS diffractometer	4944 independent reflections
Radiation source: fine-focus sealed tube	4176 reflections with *I* > 2σ(*I*)
Monochromator: graphite	*R*_int_ = 0.024
*T* = 100(2) K	θ_max_ = 25.0º
φ scans	θ_min_ = 1.5º
Absorption correction: multi-scan(Blessing, 1995)	*h* = −24→24
*T*_min_ = 0.772, *T*_max_ = 0.888	*k* = −20→20
14173 measured reflections	*l* = −18→13

### Refinement

**Table:**

Refinement on *F*^2^	Secondary atom site location: difference Fourier map
Least-squares matrix: full	Hydrogen site location: inferred from neighbouring sites
*R*[*F*^2^ > 2σ(*F*^2^)] = 0.023	H atoms treated by a mixture of independent and constrained refinement
*wR*(*F*^2^) = 0.058	*w* = 1/[σ^2^(*F*_o_^2^) + (0.0405*P*)^2^] where *P* = (*F*_o_^2^ + 2*F*_c_^2^)/3
*S* = 0.96	(Δ/σ)_max_ = 0.002
4944 reflections	Δρ_max_ = 0.26 e Å^−^^3^
422 parameters	Δρ_min_ = −0.24 e Å^−^^3^
8 restraints	Extinction correction: none
Primary atom site location: structure-invariant direct methods	

### Special details

**Table:**

Geometry. All esds (except the esd in the dihedral angle between two l.s. planes) are estimated using the full covariance matrix. The cell esds are taken into account individually in the estimation of esds in distances, angles and torsion angles; correlations between esds in cell parameters are only used when they are defined by crystal symmetry. An approximate (isotropic) treatment of cell esds is used for estimating esds involving l.s. planes.
Refinement. Refinement of *F*^2^ against ALL reflections. The weighted *R*-factor *wR* and goodness of fit *S* are based on *F*^2^, conventional *R*-factors *R* are based on *F*, with *F* set to zero for negative *F*^2^. The threshold expression of *F*^2^ > σ(*F*^2^) is used only for calculating *R*-factors(gt) *etc*. and is not relevant to the choice of reflections for refinement. *R*-factors based on *F*^2^ are statistically about twice as large as those based on *F*, and *R*- factors based on ALL data will be even larger.

### Fractional atomic coordinates and isotropic or equivalent isotropic displacement parameters (Å^2^)

**Table:**

	*x*	*y*	*z*	*U*_iso_*/*U*_eq_	Occ. (<1)
Ni1	0.628359 (9)	0.922783 (10)	0.160727 (12)	0.01907 (7)	
O1	0.59027 (5)	0.89847 (6)	0.28026 (7)	0.0250 (2)	
O2	0.56835 (5)	1.00471 (6)	0.20774 (7)	0.0205 (2)	
O3	0.57620 (5)	1.15539 (6)	0.30887 (7)	0.0256 (2)	
O4	0.47393 (5)	1.15505 (6)	0.24026 (7)	0.0267 (2)	
O5	0.64334 (6)	0.11065 (7)	0.46429 (8)	0.0281 (3)	
O6	0.7500	0.20819 (10)	0.5000	0.0282 (4)	
O7	0.58575 (7)	0.64296 (8)	0.02027 (9)	0.0328 (3)	0.84
O8	0.68135 (7)	0.95450 (7)	0.46349 (9)	0.0402 (3)	
O9	0.46557 (6)	1.28175 (7)	0.13492 (8)	0.0297 (3)	
O10	0.64991 (6)	1.25560 (8)	0.23434 (9)	0.0325 (3)	
O11	0.91635 (6)	1.15274 (7)	0.39001 (9)	0.0315 (3)	
O12	0.74662 (7)	1.20158 (7)	0.14242 (8)	0.0303 (3)	
N1	0.55048 (6)	0.87119 (7)	0.08692 (8)	0.0207 (3)	
N2	0.63078 (6)	0.97732 (7)	0.04548 (8)	0.0207 (3)	
N3	0.71184 (6)	0.96927 (7)	0.22727 (8)	0.0226 (3)	
N4	0.69405 (6)	0.83646 (7)	0.14858 (8)	0.0223 (3)	
C1	0.51077 (7)	0.81814 (8)	0.11347 (11)	0.0235 (3)	
H1	0.5206	0.7982	0.1702	0.028*	
C2	0.45629 (8)	0.79144 (8)	0.06154 (11)	0.0255 (3)	
H2	0.4292	0.7535	0.0818	0.031*	
C3	0.44195 (7)	0.82124 (8)	−0.02078 (11)	0.0247 (3)	
H3	0.4042	0.8046	−0.0574	0.030*	
C4	0.48269 (7)	0.87524 (8)	−0.04946 (10)	0.0226 (3)	
H4	0.4736	0.8960	−0.1059	0.027*	
C5	0.53721 (7)	0.89850 (8)	0.00602 (10)	0.0200 (3)	
C6	0.58367 (7)	0.95639 (8)	−0.01853 (10)	0.0202 (3)	
C7	0.57885 (7)	0.98896 (9)	−0.10007 (10)	0.0236 (3)	
H5	0.5466	0.9720	−0.1450	0.028*	
C8	0.62181 (8)	1.04655 (9)	−0.11477 (10)	0.0253 (3)	
H6	0.6193	1.0698	−0.1700	0.030*	
C9	0.66841 (7)	1.06993 (9)	−0.04812 (11)	0.0252 (3)	
H7	0.6976	1.1103	−0.0563	0.030*	
C10	0.67158 (7)	1.03343 (9)	0.03036 (11)	0.0244 (3)	
H8	0.7042	1.0488	0.0757	0.029*	
C11	0.71595 (8)	1.03584 (9)	0.27018 (11)	0.0265 (3)	
H9	0.6785	1.0677	0.2665	0.032*	
C12	0.77287 (8)	1.05973 (9)	0.31965 (11)	0.0304 (4)	
H10	0.7746	1.1075	0.3489	0.036*	
C13	0.82697 (8)	1.01303 (10)	0.32572 (12)	0.0322 (4)	
H11	0.8663	1.0278	0.3602	0.039*	
C14	0.82343 (8)	0.94467 (10)	0.28125 (11)	0.0303 (4)	
H12	0.8604	0.9120	0.2843	0.036*	
C15	0.76521 (7)	0.92429 (9)	0.23206 (10)	0.0234 (3)	
C16	0.75608 (7)	0.85144 (9)	0.18396 (10)	0.0245 (3)	
C17	0.80642 (8)	0.80069 (10)	0.17641 (11)	0.0313 (4)	
H13	0.8499	0.8129	0.2000	0.038*	
C18	0.79249 (8)	0.73202 (11)	0.13413 (11)	0.0352 (4)	
H14	0.8263	0.6963	0.1287	0.042*	
C19	0.72896 (8)	0.71589 (10)	0.09983 (11)	0.0306 (4)	
H15	0.7182	0.6686	0.0714	0.037*	
C20	0.68131 (8)	0.76983 (9)	0.10772 (10)	0.0246 (3)	
H16	0.6378	0.7591	0.0831	0.030*	
C21	0.56180 (7)	0.96360 (8)	0.27220 (10)	0.0204 (3)	
C22	0.52201 (7)	0.98935 (9)	0.33836 (10)	0.0226 (3)	
H17	0.5089	0.9524	0.3771	0.027*	
C23	0.50346 (7)	1.06176 (9)	0.34656 (10)	0.0230 (3)	
H18	0.4776	1.0720	0.3909	0.028*	
C24	0.51928 (7)	1.12879 (8)	0.29294 (10)	0.0218 (3)	
H52	0.6229 (12)	0.1235 (13)	0.4146 (18)	0.055 (5)*	
H51	0.6734 (12)	0.1389 (14)	0.4736 (16)	0.055 (5)*	
H61	0.7462 (12)	0.2357 (14)	0.5434 (16)	0.062 (7)*	
H71	0.6121 (9)	0.6122 (11)	0.0009 (13)	0.054 (6)*	0.84
H72	0.5709 (11)	0.6701 (12)	−0.0230 (9)	0.054 (6)*	0.84
H81A	0.667 (2)	0.9385 (12)	0.4132 (10)	0.055 (5)*	0.50
H82	0.6690 (9)	1.0009 (4)	0.4637 (13)	0.055 (5)*	
H81B	0.7190 (10)	0.9546 (11)	0.493 (3)	0.055 (5)*	0.50
H91	0.4719 (11)	1.2409 (14)	0.1687 (15)	0.050 (4)*	
H92	0.4483 (11)	1.3135 (14)	0.1627 (15)	0.050 (4)*	
H101	0.6230 (13)	1.2265 (15)	0.2512 (17)	0.064 (5)*	
H102	0.6288 (12)	1.2854 (15)	0.1970 (18)	0.064 (5)*	
H111	0.9073 (12)	1.1424 (14)	0.4391 (18)	0.062 (5)*	
H112	0.9532 (13)	1.1749 (14)	0.3941 (17)	0.062 (5)*	
H121	0.7777 (12)	1.2100 (14)	0.1758 (17)	0.055 (5)*	
H122	0.7154 (12)	1.2128 (14)	0.1675 (16)	0.055 (5)*	

### Atomic displacement parameters (Å^2^)

**Table:**

	*U*^11^	*U*^22^	*U*^33^	*U*^12^	*U*^13^	*U*^23^
Ni1	0.01605 (10)	0.01944 (10)	0.02130 (11)	0.00027 (7)	0.00098 (7)	0.00162 (8)
O1	0.0240 (6)	0.0223 (5)	0.0295 (6)	0.0044 (4)	0.0059 (5)	0.0055 (4)
O2	0.0196 (5)	0.0193 (5)	0.0223 (6)	−0.0006 (4)	0.0012 (4)	0.0024 (4)
O3	0.0251 (6)	0.0219 (5)	0.0287 (6)	−0.0042 (4)	−0.0004 (5)	−0.0007 (4)
O4	0.0260 (6)	0.0246 (5)	0.0281 (6)	0.0030 (5)	−0.0016 (5)	0.0013 (5)
O5	0.0238 (6)	0.0274 (6)	0.0314 (7)	−0.0012 (5)	−0.0026 (5)	0.0015 (5)
O6	0.0287 (9)	0.0286 (9)	0.0267 (9)	0.000	0.0019 (7)	0.000
O7	0.0377 (8)	0.0322 (8)	0.0288 (8)	−0.0041 (6)	0.0058 (6)	−0.0013 (6)
O8	0.0458 (8)	0.0254 (6)	0.0512 (9)	0.0005 (6)	0.0130 (7)	−0.0007 (6)
O9	0.0303 (6)	0.0230 (6)	0.0376 (7)	0.0048 (5)	0.0117 (5)	0.0022 (5)
O10	0.0241 (6)	0.0392 (7)	0.0344 (7)	−0.0042 (5)	0.0042 (5)	0.0063 (6)
O11	0.0284 (6)	0.0277 (6)	0.0388 (7)	−0.0033 (5)	0.0059 (5)	−0.0022 (5)
O12	0.0302 (7)	0.0339 (6)	0.0270 (6)	0.0017 (5)	0.0037 (5)	−0.0025 (5)
N1	0.0191 (6)	0.0175 (6)	0.0257 (7)	0.0019 (5)	0.0040 (5)	−0.0004 (5)
N2	0.0163 (6)	0.0220 (6)	0.0241 (7)	0.0014 (5)	0.0031 (5)	0.0006 (5)
N3	0.0197 (6)	0.0257 (7)	0.0224 (7)	−0.0014 (5)	0.0021 (5)	0.0037 (5)
N4	0.0202 (6)	0.0247 (6)	0.0219 (7)	0.0013 (5)	0.0025 (5)	0.0032 (5)
C1	0.0248 (8)	0.0182 (7)	0.0275 (8)	0.0003 (6)	0.0030 (6)	0.0014 (6)
C2	0.0248 (8)	0.0175 (7)	0.0344 (9)	−0.0024 (6)	0.0045 (7)	−0.0011 (6)
C3	0.0220 (8)	0.0206 (7)	0.0302 (9)	−0.0010 (6)	−0.0010 (6)	−0.0064 (6)
C4	0.0243 (8)	0.0203 (7)	0.0227 (8)	0.0023 (6)	0.0017 (6)	−0.0032 (6)
C5	0.0198 (7)	0.0167 (7)	0.0237 (8)	0.0034 (6)	0.0041 (6)	−0.0011 (6)
C6	0.0172 (7)	0.0208 (7)	0.0229 (8)	0.0039 (6)	0.0034 (6)	−0.0019 (6)
C7	0.0220 (8)	0.0255 (8)	0.0234 (8)	0.0020 (6)	0.0028 (6)	−0.0010 (6)
C8	0.0258 (8)	0.0267 (8)	0.0247 (8)	0.0051 (6)	0.0077 (6)	0.0045 (6)
C9	0.0188 (7)	0.0253 (8)	0.0326 (9)	0.0010 (6)	0.0070 (6)	0.0038 (7)
C10	0.0163 (7)	0.0271 (8)	0.0299 (9)	−0.0010 (6)	0.0035 (6)	0.0020 (6)
C11	0.0246 (8)	0.0270 (8)	0.0275 (9)	−0.0036 (6)	0.0023 (6)	0.0011 (7)
C12	0.0295 (9)	0.0302 (9)	0.0307 (9)	−0.0090 (7)	0.0018 (7)	0.0006 (7)
C13	0.0233 (8)	0.0372 (9)	0.0344 (10)	−0.0100 (7)	−0.0024 (7)	0.0036 (8)
C14	0.0200 (8)	0.0358 (9)	0.0343 (9)	−0.0029 (7)	0.0010 (7)	0.0078 (7)
C15	0.0184 (7)	0.0288 (8)	0.0229 (8)	−0.0003 (6)	0.0028 (6)	0.0068 (6)
C16	0.0207 (8)	0.0317 (8)	0.0211 (8)	0.0029 (6)	0.0031 (6)	0.0056 (6)
C17	0.0211 (8)	0.0453 (10)	0.0271 (9)	0.0077 (7)	0.0017 (7)	−0.0005 (7)
C18	0.0285 (9)	0.0462 (10)	0.0311 (10)	0.0163 (8)	0.0042 (7)	−0.0017 (8)
C19	0.0353 (9)	0.0323 (9)	0.0241 (9)	0.0088 (7)	0.0040 (7)	−0.0016 (7)
C20	0.0230 (8)	0.0269 (8)	0.0236 (8)	0.0029 (6)	0.0022 (6)	0.0016 (6)
C21	0.0156 (7)	0.0194 (7)	0.0250 (8)	−0.0029 (6)	−0.0013 (6)	0.0008 (6)
C22	0.0199 (7)	0.0231 (7)	0.0248 (8)	−0.0021 (6)	0.0031 (6)	0.0032 (6)
C23	0.0178 (7)	0.0279 (8)	0.0231 (8)	−0.0007 (6)	0.0019 (6)	−0.0015 (6)
C24	0.0238 (8)	0.0183 (7)	0.0235 (8)	0.0011 (6)	0.0039 (6)	−0.0040 (6)

### Geometric parameters (Å, °)

**Table:**

Ni1—N2	2.0461 (13)	C2—C3	1.384 (2)
Ni1—N4	2.0573 (13)	C2—H2	0.9500
Ni1—N1	2.0603 (12)	C3—C4	1.381 (2)
Ni1—N3	2.0608 (13)	C3—H3	0.9500
Ni1—O2	2.0935 (10)	C4—C5	1.389 (2)
Ni1—O1	2.1678 (11)	C4—H4	0.9500
Ni1—C21	2.4700 (16)	C5—C6	1.482 (2)
O1—C21	1.2803 (18)	C6—C7	1.388 (2)
O2—C21	1.2604 (19)	C7—C8	1.383 (2)
O3—C24	1.2604 (18)	C7—H5	0.9500
O4—C24	1.2500 (19)	C8—C9	1.383 (2)
O5—H52	0.86 (3)	C8—H6	0.9500
O5—H51	0.79 (2)	C9—C10	1.377 (2)
O6—H61	0.84 (2)	C9—H7	0.9500
O7—H71	0.8500 (11)	C10—H8	0.9500
O7—H72	0.8500 (10)	C11—C12	1.384 (2)
O8—H81A	0.8500 (10)	C11—H9	0.9500
O8—H82	0.850 (9)	C12—C13	1.379 (2)
O8—H81B	0.8500 (11)	C12—H10	0.9500
O9—H91	0.89 (2)	C13—C14	1.379 (2)
O9—H92	0.82 (2)	C13—H11	0.9500
O10—H101	0.82 (3)	C14—C15	1.387 (2)
O10—H102	0.86 (3)	C14—H12	0.9500
O11—H111	0.83 (3)	C15—C16	1.478 (2)
O11—H112	0.85 (3)	C16—C17	1.386 (2)
O12—H121	0.79 (3)	C17—C18	1.382 (3)
O12—H122	0.82 (3)	C17—H13	0.9500
N1—C1	1.342 (2)	C18—C19	1.381 (2)
N1—C5	1.346 (2)	C18—H14	0.9500
N2—C10	1.336 (2)	C19—C20	1.382 (2)
N2—C6	1.3509 (19)	C19—H15	0.9500
N3—C11	1.340 (2)	C20—H16	0.9500
N3—C15	1.350 (2)	C21—C22	1.478 (2)
N4—C20	1.337 (2)	C22—C23	1.334 (2)
N4—C16	1.354 (2)	C22—H17	0.9500
C1—C2	1.380 (2)	C23—C24	1.503 (2)
C1—H1	0.9500	C23—H18	0.9500
			
N2—Ni1—N4	99.49 (5)	N2—C6—C5	114.95 (13)
N2—Ni1—N1	79.66 (5)	C7—C6—C5	123.34 (13)
N4—Ni1—N1	96.01 (5)	C8—C7—C6	118.90 (14)
N2—Ni1—N3	98.21 (5)	C8—C7—H5	120.5
N4—Ni1—N3	79.37 (5)	C6—C7—H5	120.5
N1—Ni1—N3	174.58 (5)	C7—C8—C9	119.24 (15)
N2—Ni1—O2	94.45 (4)	C7—C8—H6	120.4
N4—Ni1—O2	164.87 (5)	C9—C8—H6	120.4
N1—Ni1—O2	92.25 (4)	C10—C9—C8	118.66 (15)
N3—Ni1—O2	92.88 (5)	C10—C9—H7	120.7
N2—Ni1—O1	155.05 (4)	C8—C9—H7	120.7
N4—Ni1—O1	104.97 (4)	N2—C10—C9	122.86 (15)
N1—Ni1—O1	92.82 (5)	N2—C10—H8	118.6
N3—Ni1—O1	91.13 (5)	C9—C10—H8	118.6
O2—Ni1—O1	61.86 (4)	N3—C11—C12	122.25 (15)
N2—Ni1—C21	124.77 (5)	N3—C11—H9	118.9
N4—Ni1—C21	135.74 (5)	C12—C11—H9	118.9
N1—Ni1—C21	93.03 (5)	C13—C12—C11	118.86 (16)
N3—Ni1—C21	92.27 (5)	C13—C12—H10	120.6
O2—Ni1—C21	30.67 (4)	C11—C12—H10	120.6
O1—Ni1—C21	31.19 (4)	C12—C13—C14	119.37 (15)
C21—O1—Ni1	87.54 (9)	C12—C13—H11	120.3
C21—O2—Ni1	91.41 (9)	C14—C13—H11	120.3
H52—O5—H51	106 (2)	C13—C14—C15	119.04 (15)
H71—O7—H72	104.48 (17)	C13—C14—H12	120.5
H81A—O8—H82	104.49 (17)	C15—C14—H12	120.5
H81A—O8—H81B	133 (4)	N3—C15—C14	121.68 (15)
H82—O8—H81B	104.49 (17)	N3—C15—C16	115.21 (13)
H91—O9—H92	106 (2)	C14—C15—C16	123.08 (14)
H101—O10—H102	107 (2)	N4—C16—C17	121.64 (15)
H111—O11—H112	110 (2)	N4—C16—C15	115.06 (13)
H121—O12—H122	105 (2)	C17—C16—C15	123.28 (14)
C1—N1—C5	118.71 (13)	C18—C17—C16	119.06 (15)
C1—N1—Ni1	126.51 (11)	C18—C17—H13	120.5
C5—N1—Ni1	114.63 (10)	C16—C17—H13	120.5
C10—N2—C6	118.56 (13)	C19—C18—C17	119.32 (15)
C10—N2—Ni1	126.10 (10)	C19—C18—H14	120.3
C6—N2—Ni1	115.21 (10)	C17—C18—H14	120.3
C11—N3—C15	118.78 (13)	C18—C19—C20	118.70 (16)
C11—N3—Ni1	125.98 (10)	C18—C19—H15	120.7
C15—N3—Ni1	115.03 (10)	C20—C19—H15	120.7
C20—N4—C16	118.60 (13)	N4—C20—C19	122.64 (15)
C20—N4—Ni1	126.33 (10)	N4—C20—H16	118.7
C16—N4—Ni1	115.04 (10)	C19—C20—H16	118.7
N1—C1—C2	122.55 (15)	O2—C21—O1	119.19 (14)
N1—C1—H1	118.7	O2—C21—C22	121.17 (13)
C2—C1—H1	118.7	O1—C21—C22	119.65 (13)
C1—C2—C3	118.46 (15)	O2—C21—Ni1	57.92 (8)
C1—C2—H2	120.8	O1—C21—Ni1	61.27 (8)
C3—C2—H2	120.8	C22—C21—Ni1	179.05 (11)
C4—C3—C2	119.73 (14)	C23—C22—C21	123.46 (14)
C4—C3—H3	120.1	C23—C22—H17	118.3
C2—C3—H3	120.1	C21—C22—H17	118.3
C3—C4—C5	118.55 (15)	C22—C23—C24	126.77 (14)
C3—C4—H4	120.7	C22—C23—H18	116.6
C5—C4—H4	120.7	C24—C23—H18	116.6
N1—C5—C4	121.97 (14)	O4—C24—O3	126.46 (14)
N1—C5—C6	115.38 (13)	O4—C24—C23	116.96 (13)
C4—C5—C6	122.63 (14)	O3—C24—C23	116.47 (13)
N2—C6—C7	121.69 (14)		
			
N2—Ni1—O1—C21	−19.88 (15)	C3—C4—C5—N1	−1.4 (2)
N4—Ni1—O1—C21	171.82 (8)	C3—C4—C5—C6	−179.67 (13)
N1—Ni1—O1—C21	−91.19 (8)	C10—N2—C6—C7	3.2 (2)
N3—Ni1—O1—C21	92.52 (8)	Ni1—N2—C6—C7	179.46 (11)
O2—Ni1—O1—C21	−0.15 (8)	C10—N2—C6—C5	−175.00 (13)
N2—Ni1—O2—C21	171.94 (8)	Ni1—N2—C6—C5	1.23 (16)
N4—Ni1—O2—C21	−31.0 (2)	N1—C5—C6—N2	−3.91 (18)
N1—Ni1—O2—C21	92.14 (9)	C4—C5—C6—N2	174.45 (13)
N3—Ni1—O2—C21	−89.59 (9)	N1—C5—C6—C7	177.89 (14)
O1—Ni1—O2—C21	0.15 (8)	C4—C5—C6—C7	−3.7 (2)
N2—Ni1—N1—C1	−178.58 (13)	N2—C6—C7—C8	−2.8 (2)
N4—Ni1—N1—C1	82.84 (12)	C5—C6—C7—C8	175.33 (13)
O2—Ni1—N1—C1	−84.47 (12)	C6—C7—C8—C9	0.2 (2)
O1—Ni1—N1—C1	−22.54 (12)	C7—C8—C9—C10	1.8 (2)
C21—Ni1—N1—C1	−53.77 (12)	C6—N2—C10—C9	−1.2 (2)
N2—Ni1—N1—C5	−3.10 (10)	Ni1—N2—C10—C9	−176.95 (11)
N4—Ni1—N1—C5	−101.68 (10)	C8—C9—C10—N2	−1.3 (2)
O2—Ni1—N1—C5	91.02 (10)	C15—N3—C11—C12	−0.5 (2)
O1—Ni1—N1—C5	152.94 (10)	Ni1—N3—C11—C12	174.11 (12)
C21—Ni1—N1—C5	121.71 (10)	N3—C11—C12—C13	−0.7 (3)
N4—Ni1—N2—C10	−88.75 (12)	C11—C12—C13—C14	1.2 (3)
N1—Ni1—N2—C10	176.81 (13)	C12—C13—C14—C15	−0.6 (3)
N3—Ni1—N2—C10	−8.24 (13)	C11—N3—C15—C14	1.1 (2)
O2—Ni1—N2—C10	85.32 (12)	Ni1—N3—C15—C14	−174.01 (12)
O1—Ni1—N2—C10	102.70 (15)	C11—N3—C15—C16	179.07 (14)
C21—Ni1—N2—C10	90.32 (12)	Ni1—N3—C15—C16	3.92 (17)
N4—Ni1—N2—C6	95.35 (10)	C13—C14—C15—N3	−0.6 (2)
N1—Ni1—N2—C6	0.91 (10)	C13—C14—C15—C16	−178.39 (15)
N3—Ni1—N2—C6	175.86 (10)	C20—N4—C16—C17	2.0 (2)
O2—Ni1—N2—C6	−90.58 (10)	Ni1—N4—C16—C17	−176.08 (12)
O1—Ni1—N2—C6	−73.20 (15)	C20—N4—C16—C15	−176.69 (14)
C21—Ni1—N2—C6	−85.58 (11)	Ni1—N4—C16—C15	5.19 (17)
N2—Ni1—N3—C11	86.12 (13)	N3—C15—C16—N4	−6.0 (2)
N4—Ni1—N3—C11	−175.69 (14)	C14—C15—C16—N4	171.85 (15)
O2—Ni1—N3—C11	−8.79 (13)	N3—C15—C16—C17	175.24 (15)
O1—Ni1—N3—C11	−70.67 (13)	C14—C15—C16—C17	−6.9 (2)
C21—Ni1—N3—C11	−39.49 (13)	N4—C16—C17—C18	−2.2 (3)
N2—Ni1—N3—C15	−99.13 (11)	C15—C16—C17—C18	176.46 (16)
N4—Ni1—N3—C15	−0.95 (11)	C16—C17—C18—C19	0.6 (3)
O2—Ni1—N3—C15	165.95 (11)	C17—C18—C19—C20	1.1 (3)
O1—Ni1—N3—C15	104.07 (11)	C16—N4—C20—C19	−0.3 (2)
C21—Ni1—N3—C15	135.25 (11)	Ni1—N4—C20—C19	177.56 (12)
N2—Ni1—N4—C20	−83.75 (13)	C18—C19—C20—N4	−1.2 (2)
N1—Ni1—N4—C20	−3.27 (13)	Ni1—O2—C21—O1	−0.25 (13)
N3—Ni1—N4—C20	179.59 (14)	Ni1—O2—C21—C22	179.69 (12)
O2—Ni1—N4—C20	119.46 (18)	Ni1—O1—C21—O2	0.25 (13)
O1—Ni1—N4—C20	91.28 (13)	Ni1—O1—C21—C22	−179.70 (12)
C21—Ni1—N4—C20	97.34 (14)	N2—Ni1—C21—O2	−9.80 (10)
N2—Ni1—N4—C16	94.21 (11)	N4—Ni1—C21—O2	168.89 (8)
N1—Ni1—N4—C16	174.69 (11)	N1—Ni1—C21—O2	−89.31 (8)
N3—Ni1—N4—C16	−2.45 (11)	N3—Ni1—C21—O2	91.82 (8)
O2—Ni1—N4—C16	−62.6 (2)	O1—Ni1—C21—O2	−179.75 (13)
O1—Ni1—N4—C16	−90.76 (11)	N2—Ni1—C21—O1	169.95 (8)
C21—Ni1—N4—C16	−84.70 (12)	N4—Ni1—C21—O1	−11.36 (11)
C5—N1—C1—C2	−1.1 (2)	N1—Ni1—C21—O1	90.44 (8)
Ni1—N1—C1—C2	174.22 (11)	N3—Ni1—C21—O1	−88.43 (8)
N1—C1—C2—C3	−0.6 (2)	O2—Ni1—C21—O1	179.75 (13)
C1—C2—C3—C4	1.3 (2)	O2—C21—C22—C23	−16.2 (2)
C2—C3—C4—C5	−0.3 (2)	O1—C21—C22—C23	163.78 (14)
C1—N1—C5—C4	2.1 (2)	C21—C22—C23—C24	−0.6 (2)
Ni1—N1—C5—C4	−173.74 (11)	C22—C23—C24—O4	107.45 (18)
C1—N1—C5—C6	−179.51 (12)	C22—C23—C24—O3	−76.2 (2)
Ni1—N1—C5—C6	4.63 (15)		

### Hydrogen-bond geometry (Å, °)

**Table:**

*D*—H···*A*	*D*—H	H···*A*	*D*···*A*	*D*—H···*A*
O5—H51···O6	0.79 (2)	1.99 (3)	2.7864 (16)	178 (3)
O5—H52···O3^i^	0.86 (3)	1.88 (3)	2.7466 (17)	177 (2)
O6—H61···O12^ii^	0.84 (2)	1.90 (2)	2.7384 (16)	174 (3)
O7—H71···O8^iii^	0.8500 (11)	1.997 (3)	2.844 (2)	174 (2)
O7—H72···O9^iv^	0.8500 (10)	1.996 (4)	2.8388 (19)	171 (2)
O8—H81A···O1	0.8500 (10)	2.539 (14)	3.3576 (18)	162 (4)
O8—H82···O5^v^	0.850 (9)	1.991 (9)	2.8406 (17)	178 (2)
O8—H81B···O8^vi^	0.8500 (11)	2.084 (11)	2.918 (3)	167 (4)
O9—H91···O4	0.89 (2)	1.87 (3)	2.7516 (16)	173 (2)
O9—H92···O1^vii^	0.82 (2)	1.96 (3)	2.7701 (16)	173 (2)
O10—H101···O3	0.82 (3)	1.88 (3)	2.6905 (17)	168 (3)
O10—H102···O11^viii^	0.86 (3)	1.89 (3)	2.7409 (19)	177 (3)
O11—H111···O5^ix^	0.83 (3)	2.03 (3)	2.8333 (19)	161 (2)
O11—H112···O9^viii^	0.85 (3)	1.95 (3)	2.7752 (18)	162 (2)
O12—H121···O10^viii^	0.79 (3)	2.00 (3)	2.7844 (18)	172 (2)
O12—H122···O10	0.82 (3)	1.97 (3)	2.7839 (18)	170 (2)
C23—H18···O7^vii^	0.95	2.38	3.288 (2)	159

Symmetry codes: (i) *x*, *y*−1, *z*; (ii) *x*, −*y*+3/2, *z*+1/2; (iii) *x*, −*y*+3/2, *z*−1/2; (iv) −*x*+1, −*y*+2, −*z*; (v) *x*, *y*+1, *z*; (vi) −*x*+3/2, *y*, −*z*+1; (vii) −*x*+1, *y*+1/2, −*z*+1/2; (viii) −*x*+3/2, −*y*+5/2, −*z*+1/2; (ix) −*x*+3/2, *y*+1, −*z*+1.
